# Dicer Is Required for Haploid Male Germ Cell Differentiation in Mice

**DOI:** 10.1371/journal.pone.0024821

**Published:** 2011-09-16

**Authors:** Hanna M. Korhonen, Oliver Meikar, Ram Prakash Yadav, Marilena D. Papaioannou, Yannick Romero, Matteo Da Ros, Pedro L. Herrera, Jorma Toppari, Serge Nef, Noora Kotaja

**Affiliations:** 1 Department of Physiology, Institute of Biomedicine, University of Turku, Turku, Finland; 2 Department of Genetic Medicine and Development, University of Geneva Medical School, Geneva, Switzerland; 3 Department of Cell Physiology and Metabolism, University of Geneva Medical School, Geneva, Switzerland; 4 Department of Pediatrics, University of Turku, Turku, Finland; Institute of Genetics and Molecular and Cellular Biology, France

## Abstract

**Background:**

The RNase III endonuclease Dicer is an important regulator of gene expression that processes microRNAs (miRNAs) and small interfering RNAs (siRNAs). The best-characterized function of miRNAs is gene repression at the post-transcriptional level through the pairing with mRNAs of protein-encoding genes. Small RNAs can also act at the transcriptional level by controlling the epigenetic status of chromatin. Dicer and other mediators of small RNA pathways are present in mouse male germ cells, and several miRNAs and endogenous siRNAs are expressed in the testis, suggesting that Dicer-dependent small RNAs are involved in the control of the precisely timed and highly organised process of spermatogenesis.

**Principal Findings:**

Being interested in the Dicer-mediated functions during spermatogenesis, we have analysed here a male germ cell-specific *Dicer1* knockout mouse model, in which the deletion of *Dicer1* takes place during early postnatal development in spermatogonia. We found that *Dicer1* knockout testes were reduced in size and spermatogenesis within the seminiferous tubules was disrupted. *Dicer1* knockout epididymides contained very low number of mature sperm with pronounced morphological abnormalities. Spermatogonial differentiation appeared unaffected. However, the number of haploid cells was decreased in knockout testes, and an increased number of apoptotic spermatocytes was observed. The most prominent defects were found during late haploid differentiation, and Dicer was demonstrated to be critical for the normal organization of chromatin and nuclear shaping of elongating spermatids.

**Conclusions/Significance:**

We demonstrate that Dicer and Dicer-dependent small RNAs are imperative regulators of haploid spermatid differentiation and essential for male fertility.

## Introduction

Spermatogenesis is under strict gene control that governs the precisely timed events leading to the production of mature spermatozoa capable of fertilization [1], [2]. It includes proliferation, differentiation and morphogenesis of male germ cells [3]. The process begins when diploid spermatogonia multiply by consecutive mitotic divisions and then enter the meiotic program, which involves chromosome duplication, homologous chromosome pairing, synaptonemal complex formation, meiotic recombination and meiotic divisions resulting in the formation of haploid round spermatids. Haploid germ cells then undergo a dramatic differentiation phase, spermiogenesis, which includes acrosome and flagellum formation, nuclear reshaping and massive chromatin reorganization during which histones are replaced by testis-specific proteins called protamines [4]. The histone-protamine transition sets limitations to the male germ cell–specific gene expression since protamine-bound genes are largely silenced. Therefore, post-transcriptional mRNA control is active in late spermatogenic cells to ensure the correct timing of protein expression and to provide mRNAs in transcriptionally inactive elongating spermatids.

Small non-coding RNAs are crucial gene regulators that can target gene expression both post-transcriptionally by mRNA silencing and transcriptionally by mediating changes in chromatin organization [5]. Small RNAs are also important regulators of male fertility, and distinct classes with different mechanisms of biogenesis and function have been found in the male germ line [6]. One of these classes consists of microRNAs (miRNAs), several of which are expressed in spermatogenic cells, implying that they have an important role in gene regulation during spermatogenesis [7], [8]. miRNAs mostly act by destabilizing target mRNAs or inhibiting their translation [9]. Each of them may target hundreds of distinct mRNAs and thus expression of most of protein-coding genes is controlled by these small regulatory RNAs [10]. PIWI-interacting RNAs (piRNAs) are predominantly expressed in the germ cell lineage. They are synthesized in large quantities and their functions include silencing of transposon expression [11]. Processing mechanisms for piRNAs have not yet been identified but their synthesis does not involve the RNase III endonuclease Dicer that is critical for production of miRNAs and small interfering RNAs (siRNAs) [6], [12]. miRNA processing from hairpin-loop-folded primary precursors requires two RNase III–like enzymes, Drosha and Dicer [13]. In contrast, double-stranded siRNA precursors can be processed by Dicer alone, which emphasizes the diversity of small RNA pathways. siRNA precursors are usually introduced in cells exogenously, for example by viruses, but as originally described in plants and nematodes, endogenous siRNAs (endo-siRNAs) can also be produced and can have important functions in gene silencing [6]. There is increasing evidence that endo-siRNAs can be used as a gene control mechanism also in mammals [6], [14]–[16].

Dicer is vital for mouse embryogenesis since its deletion results in an early embryonic lethal phenotype [17]. The importance of Dicer in several differentiation programs has been demonstrated, including mouse female and male germ cell maturation [18]–[21]. In the mouse testis, Sertoli cell–specific deletion of *Dicer1* revealed its crucial importance for the normal function of these somatic nursing cells in supporting male germ cell differentiation [22], [23]. The role of intrinsic miRNA pathways in male germ cells has been studied using a mouse model with a specific deletion of *Dicer1* in primordial germ cells (PGCs) induced by a *TNAP-Cre* transgene [20], [21]. These studies demonstrated the defects in PGC proliferation and spermatogenesis, thus suggesting the potential importance of Dicer-dependent pathways on postnatal male germ cell differentiation [21]. However, these studies could not address the exact role of Dicer in adult spermatogenesis since *Dicer1* was already depleted in PGCs at embryonic day 10 [24], and thus the development of embryonic germ cells was interfered. Phenotypic analysis of this mouse line was also problematic due to the low penetrance of *TNAP-Cre* transgene. Therefore, different fully penetrant mouse lines in which *Dicer1* deletion occurs in postnatal male germ cells are required to assess Dicer-mediated functions in adult spermatogenesis. We generated a knockout mouse model, in which *Dicer1* was deleted specifically in spermatogonia to demonstrate the role of Dicer and Dicer-dependent small RNAs in the regulation of postnatal male germ cell development. We found that *Dicer1* deletion caused a failure in haploid differentiation resulting in abnormal spermatozoa and eventual male infertility.

## Results

### Expression of *Dicer1* during spermatogenesis

**Figure 1:**
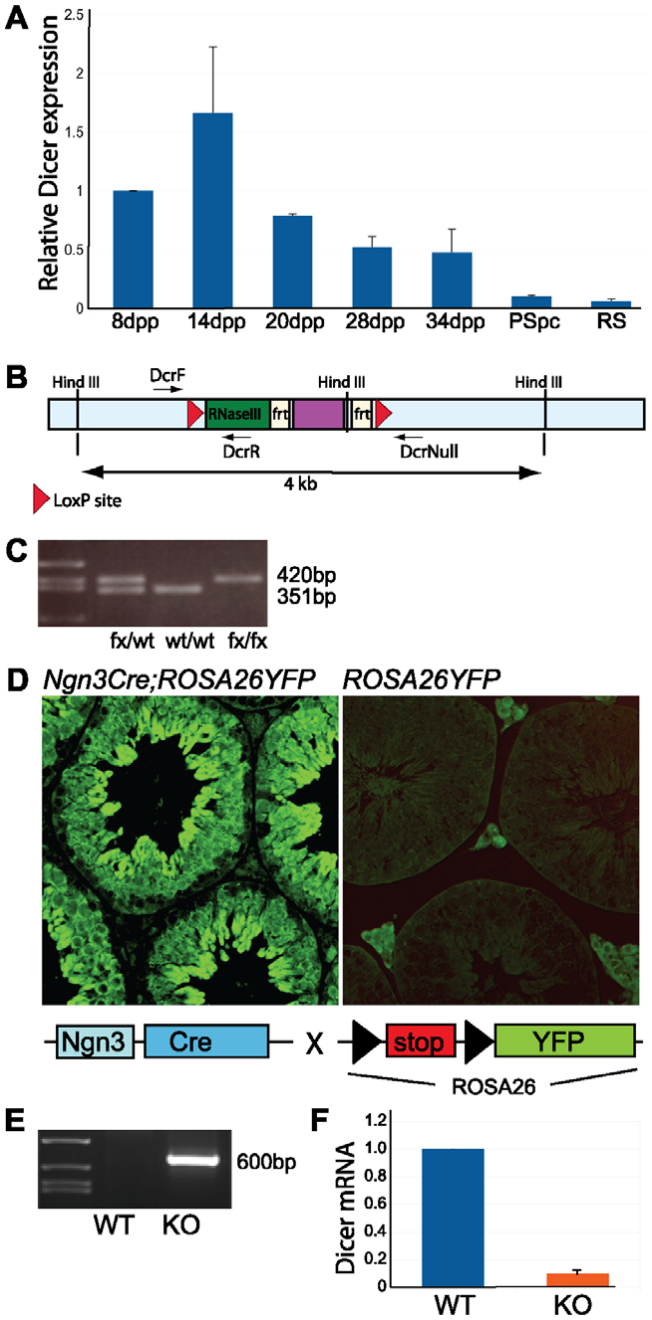
Generation of a germ cell-specific *Dicer1* knockout mouse line. A) Expression of *Dicer1* mRNA as analyzed by RT-PCR on total testis RNA extracted from different time points during the first wave of spermatogenesis at 8, 14, 20, 28 and 34 dpp, and from isolated pachytene spermatocytes (PSpc) and round spermatids (RS). B) Schematic representation of the floxed *Dicer1* allele, in which two loxP-sites are flanking the exon that encodes the second RNase III domain of Dicer. C) The floxed allele was genotyped using primers DcrF and DrcR, yielding a 420 bp band for the floxed (fx) and a 351 bp band for the wild type (wt) allele. D) To assess the Cre-mediated recombination efficiency, Ngn3Cre mice were crossed with *ROSA26YFP* reporter mice. Anti-GFP immunostaining revealed that *Ngn3Cre;ROSA26YFP* testes sections show strong GFP expression in germ cells, whereas control *ROSA26YFP* littermates do not. Note that Leydig cells are GFP positive due to autofluorescence. E) Genomic PCR on the whole testis DNA with DcrF and DcrNull primers confirmed the deletion of *Dicer1* at postnatal day 10. F) *Dicer1* mRNA is dramatically decreased in knockout testes. RT-PCR analysis of the testes of 9 weeks old mice using primers for *Dicer1* mRNA. The reverse primer is located in the deleted exon and no PCR product is produced from cells with deleted *Dicer1* alleles. KO: *Dcr(fx/fx);Ngn3Cre*, WT: *Dcr(fx/fx)*.

*Dicer1* is known to be expressed in the mouse testis [25], [26], but its exact expression pattern is unclear. Due to the importance of Dicer-dependent pathways in various cellular processes, Dicer is considered to be constitutively expressed in all cell types. However, gene expression patterns during spermatogenesis are usually strictly controlled both spatially and temporally [1], and we wanted to examine whether *Dicer1* expression is differentially regulated during the progress of spermatogenesis. We performed quantitative RT-PCR analysis of juvenile mouse testes to show the time course of *Dicer1* mRNA appearance (Fig. 1A). Total testis RNA was extracted from wild type mice sacrificed at different time points during the synchronized first wave of spermatogenesis. These samples thus represent different phases of germ cell differentiation within the seminiferous epithelium. Testis collected at 8 days post partum (dpp) contains spermatogenic cells just prior to or at the onset of meiosis; at 14 dpp pachytene spermatocytes appear, at 20 dpp round spermatids have already been generated, and at 28 dpp the elongation of spermatids has started. *Dicer1* expression was highest in testes collected at 14 dpp, and the amount of *Dicer1* mRNA decreased in the testes of older animals (Fig. 1A). This indicates that *Dicer1* is expressed at higher levels in early germ cell types such as spermatogonia and early spermatocytes than in late spermatocytes and spermatids as the appearance of these cell types in the testis dilutes the *Dicer1* mRNA signal. *Dicer1* mRNA could be detected in isolated pachytene spermatocytes and round spermatids, but at relatively low levels, further supporting the results obtained from the analysis of juvenile testes (Fig. 1A).

### Germ cell-specific deletion of *Dicer1* in the mouse testis

To generate a mouse line with a specific deletion of *Dicer1* in postnatal male germ cells, we crossed a mouse line carrying a floxed *Dicer1* allele (*Dcr(fx/fx)*) with a transgenic mouse line expressing the Cre recombinase under the control of a Neurogenin3 (*Ngn3*) promoter *(Ngn3Cre)* [27]. In this study, we used a floxed *Dicer1* allele that was generated by inserting two loxP sites around exon 24 that encodes most of the second RNase III domain (Fig. 1B,C) [28]. Cre-dependent recombination generates a mutated allele that encodes a truncated Dicer protein still recognized by an antibody made against a region at the C-terminus of the protein [28].

Ngn3 is endogenously expressed in male germ cells starting from day 5 dpp, and it has been shown to be expressed in type A spermatogonia that give rise to all differentiating germ cells [29]. Thus *Ngn3*-promoter-driven Cre is produced at early stage of postnatal development and induces *Dicer1* deletion in spermatogonia. To verify the Cre-mediated recombination in spermatogenic cells, the *Ngn3Cre* line was crossed with a transgenic mouse line expressing a Cre-recombination sensitive *ROSA26YFP* reporter gene (Fig. 1D) [30]. Analysis of the testes from *Ngn3Cre; ROSA26YFP* mice demonstrated the Cre-dependent YFP expression in differentiating male germ cells throughout the meiotic and postmeiotic stages (Fig. 1D). *Dcr(fx/fx); Ngn3Cre* mice with germ cell-specific knockout of both *Dicer1* alleles (hereafter referred to as *Dicer1* knockout mice) were generated by crossing *Dcr(fx/wt); Ngn3Cre* and *Dcr(fx/fx)* animals. The Cre recombination activity on *Dicer1* was demonstrated through genomic PCR using primers specifically amplifying the deleted *Dicer1* allele (Fig. 1E). A deletion-specific 600 bp band was produced in the testicular DNA from 10 dpp knockout but not wild type control mouse, demonstrating that the recombination had taken place. Importantly, amplification of *Dicer1* mRNA using one primer hybridizing within the deleted area confirmed a dramatic decrease of *Dicer1* mRNA in adult knockout testes (Fig. 1F).

### *Dicer1* deletion in postnatal male germ cells results in defective spermatogenesis and infertility

**Figure 2:**
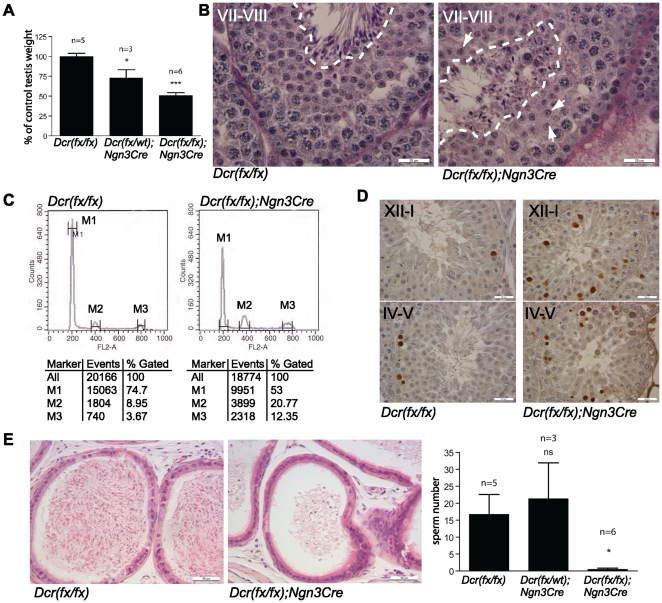
Abnormal spermatogenesis in *Dicer1* knockout mice. A) Decreased size of adult *Dcr(fx/fx);Ngn3Cre* testes as compared to *Dcr(fx/fx)* or *Dcr(fx/wt); Ngn3Cre* testes. B) PAS staining of stage VII-VIII seminiferous tubules of 8 weeks old control and knockout mice. Spermatid elongation is clearly affected as indicated by the amount, organization and nuclear morphology of the elongating spermatids. The area containing elongating spermatid is circled with white dashed line. White arrows point to the acrosomes of some selected round spermatids at different developmental steps. C) Flow cytometric analysis of germ cells isolated from knockout (right panel) and control (left panel) testes. M1, M2 and M3 populations correspond to 1C, 2C and 4C cells respectively. 74.7% of the counted cells were haploid in *Dcr(fx/fx)* testes, whereas this number appeared reduced (53%) in *Dcr(fx/fx); Ngn3Cre* animals. D) TUNEL staining revealed increased apoptosis in the seminiferous tubules of 8 weeks old knockout mice. The stages containing most apoptotic cells are the same as in the wild type but the amount of apoptotic cells per cross-section is increased in knockout testes. E) Hematoxylin-eosin staining of the cauda epididymides of *Dcr(fx/fx)* and *Dcr(fx/fx); Ngn3Cre* mice and sperm counts demonstrated the dramatically lowered amount of sperm in the knockout epididymis. Sperm number (×10^6^). Scale bars in (B) and (D): 25 µm and in (E): 50 µm.

Germ cell-specific *Dicer1* knockout mice grew normally and did not display any visible physiological or anatomical gross abnormalities in adulthood. However, when mated with the wild type C57BL/6J females, knockout males were unable to sire any pups (data not shown). The testis size of knockout animals was reduced about 50% compared to control littermates (Fig. 2A). Periodic acid-Schiff (PAS) and hematoxylin-eosin (HE) staining of knockout testis sections demonstrated a severe disruption of spermatogenesis (Fig. 2B, Fig. S1 and Fig. S2). Spermatogonia and early meiotic cells appeared normal without any prominent defects, and the general organization of Sertoli and germ cells in the seminiferous epithelium was unaffected (Fig. S3). However, compared to control littermates, less haploid cells were produced (Fig. 2C). Because of the decreased number of haploid cells in knockout testes, we decided to measure apoptotic activity in the seminiferous epithelium of knockout mice compared to that of control littermates by TUNEL assay. An increased number of apoptotic cells was detected in knockout testis (Fig. 2D). Apoptotic cells were identified as mostly spermatocytes based on their location and appearance in the seminiferous epithelium. Apoptotic cells within knockout tubules were normally found at stages XII-I, when spermatocytes that have failed meiosis are eliminated, and at stages IV-V, after the mid-pachytene check-point. However, the number of apoptotic cells in these stages was clearly increased.

The most obvious spermatogenic failure took place during haploid differentiation, as demonstrated by the abnormal organization, amount and morphology of elongating spermatids (Fig. 2B). The seminiferous epithelium is organized in a cyclic manner in twelve distinct stages, with each stage containing a specific association of different germ cell types at distinct phases of differentiation [3], [31]. Microscopic analysis of knockout testes often revealed disorganized stages of seminiferous epithelial cycle with haploid cells from different developmental stages being mixed in the same cross sections (Fig. 2B, round spermatid acrosomes are pointed by arrows). Living cell phase contrast microscopy of stage-specific pieces of seminiferous tubules [31] supported these findings (data not shown). In addition, defects in acrosomes, such as fragmented acrosomes or abnormal acrosomal vacuoles were frequently detected (Fig. S4). Histological and sperm count analysis of the cauda epididymides confirmed the spermatogenic problems by demonstrating a drastic reduction in the number of mature spermatozoa. In addition, numerous exfoliated immature germ cells were frequently detected in the epididymal lumen (Fig. 2E).

### Meiosis in *Dicer1* knockout spermatocytes

**Figure 3:**
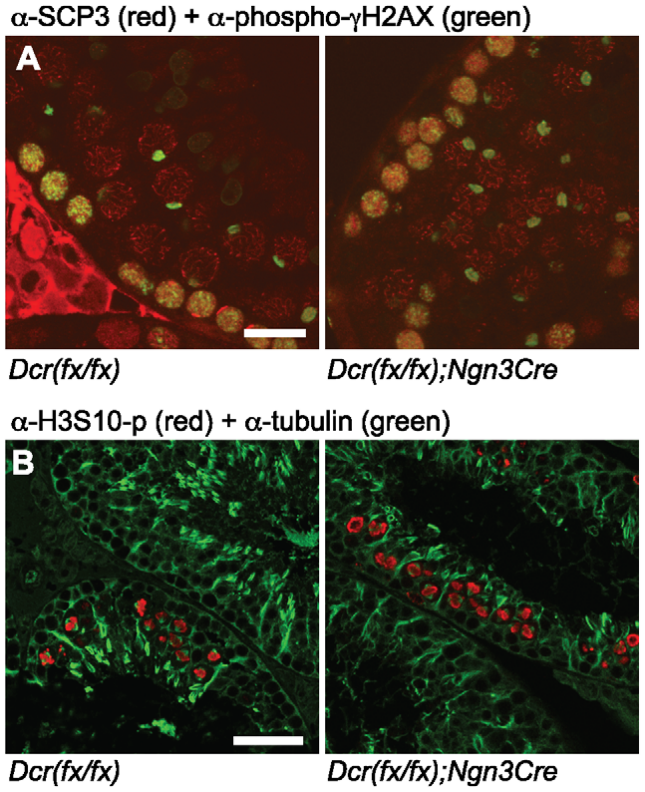
Meiotic analysis of *Dicer1* knockout testes. A) Confocal immunofluorescence microscopy of testis sections from control and knockout mice. An anti-SCP3 antibody (red) was used to detect synaptonemal complexes and an antibody against phosphorylated γH2AX (green) was used to visualize unsynapsed X and Y chromosomes in the sex body of pachytene spermatocytes. Scale bar: 20 µm. B) An antibody against phosphorylated H3 Serine 10 (red) was used to visualize meiotic metaphases. Double-staining with anti-tubulin (green) was used to visualize meiotic spindles and general organization of microtubular network in the seminiferous epithelium. Scale bar: 50 µm.

Light microscopy of knockout seminiferous tubules revealed no gross abnormalities in meiotic spermatocytes. We assessed the formation of synaptonemal complexes in knockout pachytene spermatocytes by staining with an antibody against SCP3 (Synaptonemal complex protein 3), one of the major components of the lateral elements of synaptonemal complexes. Synaptonemal complexes were normally detected, and double staining with an antibody against phosphorylated histone H2AX that is used as a marker for silenced X and Y chromosomes in the sex body, revealed the presence of sex bodies in knockout pachytene spermatocytes (Fig. 3A). Synaptonemal complexes were also detectable through electron microscopy of knockout testes (Fig. S4). Immunostaining of phosphorylated Serine 10 of histone H3, a marker of mitotic and meiotic chromatin condensation, revealed the presence of metaphase plates in the knockout tubules (Fig. 3B).

### Elongation of spermatids is disrupted in *Dicer1* knockout mice

**Figure 4:**
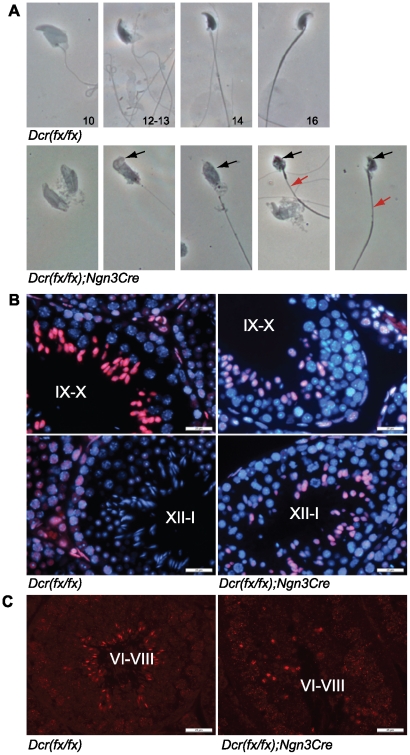
Defects in late spermiogenesis. A) Phase contrast microscopy of spermatogenic cells revealed defects in elongating spermatids. Knockout elongating spermatids had malformed head shape and abnormal chromatin condensation (black arrows). Formation of the flagellum was also affected. Red arrows point to the disorganized midpiece in elongating spermatids. B) Immunofluorescence of PFA-fixed testis sections with anti-acetylated-H3 antibody (red). Knockout tubules showed strong hyperacetylation of H3 in step 9–10 spermatids, but the signal was retained in later stages most probably indicating an arrest or delay in spermatid elongation. Nuclei are stained with DAPI (blue). C) Immunofluorescence of testis sections with anti-PRM1 antibody (red) demonstrated the decreased number of protamine-containing elongating spermatids in knockout tubules. Scale bar: 25 µm.

The abnormal spermiogenesis detected in knockout testis sections (Fig. 2B) prompted us to analyze the progress of haploid cell differentiation in greater detail. Phase contrast microscopy of drying down preparations demonstrated that spermatid elongation is severely affected in knockout mouse testes (Fig. 4A). Only very few (less than ten) normal appearing step 15–16 spermatids were detected among all spermatogenic cells analyzed. Nearly all late spermatids showed abnormal head shape and chromatin condensation and a disrupted organization of tail accessory structures (Fig. 4A). The chromatin condensation state of most of the elongating spermatids resembled that of step 9–10 spermatids, but some heads with condensed chromatin were also observed. Because of the observed problems in chromatin condensation, we went on to further study the status of histone acetylation. Acetylated forms of H2A, H2B, H3 and H4 appear in step 9–11 elongating spermatids, and disappear later in condensing spermatids, thus preceding the histone-protamine transition [32]. In wild type testes, H3 became hyperacetylated in step 9 spermatids, and the acetylation signal disappeared in step 11 spermatids (Fig. 4B). Hyperacetylation was detected in knockout elongating spermatids, but it was not stage-specific and nearly all the cross-sections contained elongated spermatids with hyperacetylated H3 (Fig. 4B). In addition, elongating spermatids that were positive for protamine PRM1 were also greatly reduced in knockout testes compared to control littermates (Fig. 4C). Overall, these findings suggest an arrest in elongation prior to the histone-protamine exchange.

**Figure 5:**
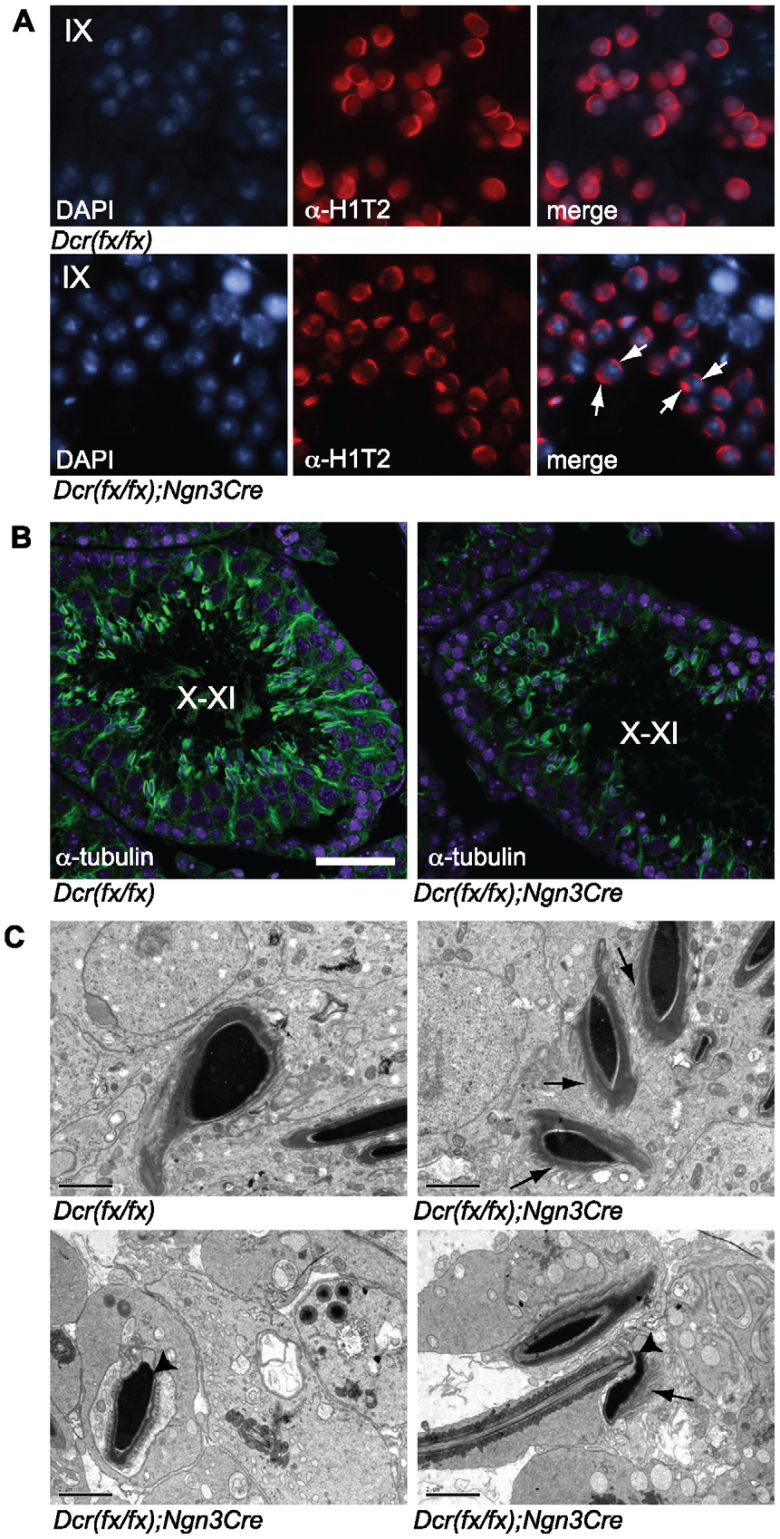
Elongating spermatids show defects in the polarization of the head and organization of the manchette. A) Immunofluorescence of testis sections with an antibody against a testis-specific histone 1 variant, H1T2. H1T2 staining in knockout step 9 spermatids (lower panel) is no longer correctly polarized beneath the developing acrosome, and is in fact localized at two opposite edges of the nucleus. White arrows in the knockout merge picture point to the bipolarized H1T2 staining of two selected step 9 spermatids. Scale bar: 20 µm. B) Fluorescence confocal microscopy of testis sections immunostained with anti-tubulin revealed the disorganized manchette structure in knockout step 9–12 elongating spermatids. Scale bar: 50 µm. C) Electron microscopy of knockout condensed elongating spermatids revealed defects in head shaping, disorganized manchettes (arrows) and abnormal bending of the head (arrowhead). Scale bar: 2 µm.

Due to the abnormal head shape of elongating spermatids, we decided to study additional proteins that are known to be critical for chromatin architecture. H1T2/H1FNT, a testis-specific histone H1 variant, has been shown to have a polarized localization within the round and elongating spermatid nucleus, being concentrated in a cap-like structure at the inner periphery of the nuclear membrane at the apical pole. H1T2 was demonstrated to confer to the spermatid nucleus the polarity that is essential for proper DNA condensation and elongation of spermatids [33]. Interestingly, the polarized localization of H1T2 was disrupted in knockout spermatids, and H1T2 showed a bipolar localization pattern at both the apical and basal side of the nucleus (Fig. 5A). Manchette is a microtubule structure in elongating spermatids that is suggested to be involved in the shaping of sperm head. Anti-tubulin immunostaining revealed a normal manchette located in an organized fashion at the basal side of control nuclei, while this was not the case in knockout elongating spermatids, in which anti-tubulin staining was distributed more randomly in the cytoplasm of spermatids, often surrounding the whole nucleus (Fig. 5B). Furthermore, electron microscopy studies of late elongating spermatids revealed disrupted microtubule organization as well as problems in nuclear shaping (Fig. 5C).

### Abnormal morphology of *Dicer1* knockout spermatozoa

**Figure 6:**
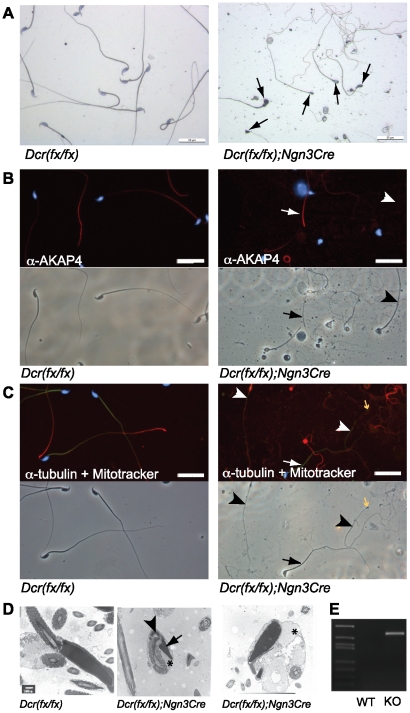
Abnormal morphology of knockout sperm. A) Very few spermatozoa could be isolated from knockout cauda epididymides, and all retrieved cells had abnormal morphology (arrows), as revealed by hematoxylin staining of the sperm slides. B) Immunofluorescence of knockout and wild type epididymal sperm with an anti-AKAP4 antibody (red) was performed to detect the fibrous sheath of the sperm flagellum. Many tails were lacking the detectable fibrous sheaths. An arrow points to a flagellum with anti-AKAP4-positive fibrous sheath, whereas an arrowhead shows a sperm tail without anti-AKAP4 staining. C) Immunostaining with an anti-tubulin antibody (red) combined with the Mitotracker staining (green) revealed the absence of mitochondrial sheath in some spermatozoa. An arrow points to the mitochondrial staining in the midpiece, and arrowheads indicate the absence or mislocalized mitochondrial sheath. Pin-head sperm with tubulin-positive axonemal structures but no DAPI-positive heads were also frequently observed (yellow arrow). Scale bar in (A), (B) and (C): 25 µm. D) Electron microscopy of epididymal sperm revealed small abnormally shaped nuclei (arrow), bending of tails from the neck region (arrowhead) and excess of cytoplasm (asterisk). Scale bar: 0.5 µm. E) Genomic PCR on the whole testis DNA with DcrF and DcrNull primers confirmed the deletion of *Dicer1* in the epididymal sperm of knockout mice.

In order to assess the morphology of *Dicer1* knockout spermatozoa, we isolated sperm from cauda epididymides of wild type and knockout mice and stained them with hematoxylin. Knockout spermatozoa were clearly abnormal with defective head structures including small heads and abnormal head shapes, as well as tail abnormalities including thin tails and disorganized accessory structures (Fig. 6A). In fact, only very few (less than ten) normal-looking spermatozoa were detected among all cells that were analyzed. Immunostaining with a fibrous sheath marker (anti-AKAP4) demonstrated that the fibrous sheath is present in some but not all of the spermatozoa (Fig. 6B). The same kind of observation was made for the mitochondrial sheath as revealed by Mitotracker staining (Fig. 6C). Furthermore, several pin-head spermatozoa with tubulin-positive thin tails but no DAPI-positive nuclei were detected (Fig. 6C). Electron microscopic examination of epididymal sperm revealed small abnormal heads that were frequently bent over the tail, and an excess of cytoplasm (Fig. 6D). To confirm the penetrance of Cre expression and the presence of *Dicer1* deletion in epididymal sperm of knockout mice, spermatozoa from the cauda epididymides were isolated and sperm DNA was examined for Cre recombination. We found that the epididymal sperm from knockout mice carried a deleted *Dicer1* allele (Fig. 6E).

### Expression of transposable elements and centromeric repeat transcripts in *Dicer1* knockout testes

**Figure 7:**
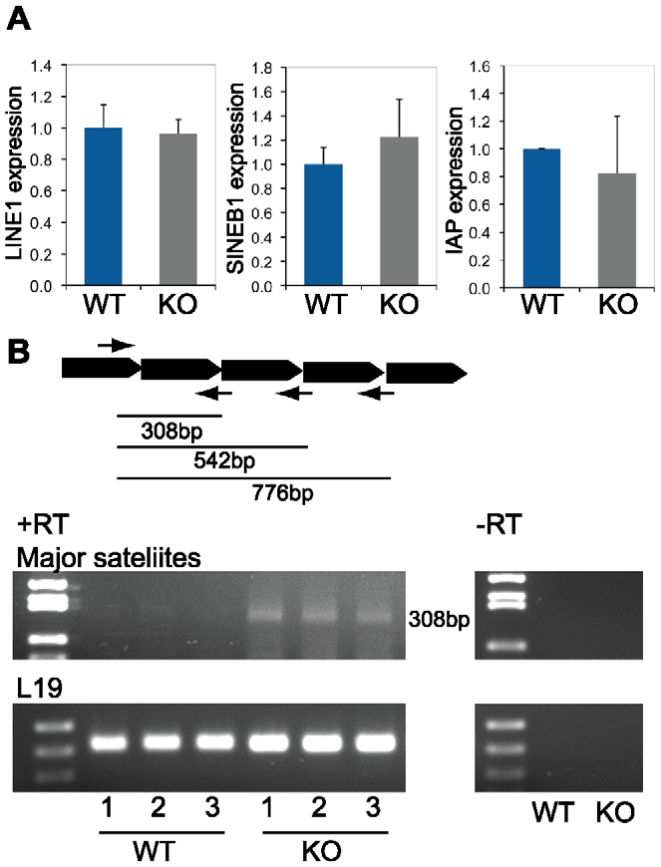
Expression of transposable elements and centromeric repeat transcripts in *Dicer1* knockout testes. A) Quantitative RT-PCR analysis of 28 days old *Dcr(fx/fx)* (WT) and *Dcr(fx/fx); Ngn3Cre* (KO) total testis RNA using LINE1, SINEB1 and IAP primers. No differences in transposon expression between control and knockout samples were revealed. B) Expression of major centromeric repeat sequences was induced in 28 days old knockout testes. A schematic diagram shows the organization of major repeats and locus-specific primers used in the study [46]. Only the 308 bp band was detected in our optimized conditions. Three experimental triplicates (1–3) are shown for both wild type and knockout reactions. As a control, reactions were performed with mock-transcribed cDNAs (-RT).

Expression of transposable elements is induced in mouse oocytes lacking Dicer [18]. In addition, human Dicer mediates the degradation of Alu/SINE transposable elements in retinal pigmented epithelium [34]. We thus decided to examine the expression of some transposable elements in the testis of knockout mice and their wild type littermates to assess weather Dicer have similar functions in male germ cells. We did not find any differences in LINE1 (Long Interspersed Nuclear Element 1), SINEB1 (Short Interspersed Nuclear Element 1), or IAP (Intracisternal A-Particle) expression in 28 days old or adult knockout testes as compared to control littermates (Fig. 7A).

Since the absence of Dicer in mouse embryonic germ cells has been shown to induce aberrant expression of centromeric transcripts [35], [36], we decided to study the status of repetitive element silencing in knockout testes. We found that the transcription of centromeric major repeats was induced in our mouse model, indicating that Dicer is required for the correct silencing of these repeat sequences (Fig. 7B). Furthermore, we wished to find out if the absence of Dicer has any effects on heterochromatin organization during spermatogenesis. The localization pattern of the centromeric protein CENP-A in the nucleus of spermatogenic cells was not drastically changed in knockout testes, suggesting that there is not general widespread disorganization of the centromeric heterochromatin areas (Fig. S5). Similar results were obtained by immunostaining using antibodies against other heterochromatin markers, such as heterochromatin protein HP-1 (data not shown), and dimethylated H3K9 (Fig. S5).

## Discussion

We have demonstrated here that Dicer is imperative for normal differentiation of haploid male germ cells in the mouse testis. To our knowledge, this is the first study of a postnatal deletion of *Dicer1*, which has enabled specific dissection of the affected spermatogenic steps without interfering with the embryonic functions of PGCs. Endogenous *Ngn3* is expressed in type A spermatogonia, which give rise to the entire panel of differentiating male germ cells [29]. *Ngn3*-promoter-driven Cre expression is fully penetrant and appears to reflect the expression of endogenous Ngn3 in spermatogonia. We should note here that Ngn3 is known to be an important regulator of pancreatic development and is expressed in pancreatic islet precursor cells [27]. In addition, *Ngn3*-promoter-driven transgenes are also expressed in brain [37]. Here, we did not analyze the pancreas and brain phenotypes of *Dcr(fx/fx); Ngn3Cre* mice, but no gross abnormalities in the development or behavior of the mice were observed. miRNAs are known to remain in the cells for some time after the deletion of *Dicer1*, and therefore we did not include spermatogonial proliferation phase in our analysis but focused on the later steps of spermatogenesis. Our results are supported by the parallel analysis of a conditional mouse model using *Ddx4*-promoter-driven expression of Cre to induce *Dicer1* deletion in male germ cells (Romero et al., co-submitted manuscript). In the *Dcr(fx/fx); Ddx4Cre* mouse line, the deletion of *Dicer1* takes place earlier than in our model and, accordingly, shows a much stronger abnormal meiotic phenotype. We believe that the reason for this difference is the later expression of the *Ngn3Cre* transgene, which might enable meiotic cells to escape the effects of *Dicer1* deletion. However, the decreased number of haploid cells and the increased number of apoptotic spermatocytes in the seminiferous epithelium of *Dcr(fx/fx); Ngn3Cre* testis supports a role of Dicer-dependent pathways in meiosis. Importantly, the haploid phenotype of both mouse lines is very similar, highlighting the central role of Dicer-dependent pathways in postmeiotic differentiation.

The most prominent spermatogenic defects in our *Dicer1* knockout mice were found during the elongation phase of haploid differentiation. Elongation appeared to be hindered before nuclear condensation had started. This was demonstrated by the high number of uncondensed elongating spermatids as detected by phase contrast microscopy of spermatogenic cell spreads (Fig. 4A), the presence of hyperacetylated H3-positive elongating spermatids in all the stages of seminiferous epithelial cycle (Fig. 4B) and correspondingly the low number of protamine-positive nuclei (Fig. 4C) in knockout testes. Some of the germ cells were able to undergo condensation as evidenced by the presence of condensed nuclei among both testicular elongating spermatids and mature sperm (Figs. 4,5,6). However, the head morphology of these cells never appeared normal and nuclei were small and misshapen; defects in the tail development were also frequently observed. Interestingly, we demonstrate that the polarized localization of histone 1 variant H1T2 was disrupted in *Dicer1* knockout mice, with H1T2 localizing on both the apical and basal sides of the nucleus. H1T2 is considered to be a molecular marker that can reveal chromatin disorganization in round and elongating spermatids [38]. *H1t2* knockout mice show delayed nuclear condensation and aberrant elongation of spermatids [33]. A bipolar H1T2 mislocalization similar to the one observed in *Dicer1* knockout spermatids, is also detected in testes devoid of Trf2, a transcriptional regulator involved in nuclear chromatin organization [38], [39]. Therefore, our results suggest that Dicer-dependent pathways are involved in the control of chromatin architecture in haploid male germ cells.

The functions mediated by small RNAs are diverse. Small RNAs in animal cells can act at the post-transcriptional level by silencing or sequestering protein-coding mRNAs or transposable element transcripts, at the chromatin level by affecting gene transcription or at the genomic level through the regulation of chromosome segregation [6]. The predominant function of mammalian miRNAs has been demonstrated to be the destabilization of target mRNAs [9]. While this manuscript was under revision, Dicer-dependent but Drosha-independent endo-siRNAs were reported in male germ line that can function in post-transcriptional control of a wide variety of protein encoding mRNAs [16]. Therefore, it is likely that at least some of the defects in *Dicer1* knockout testes are due to the absence of miRNA and/or endo-siRNA-mediated mRNA regulation. Post-transcriptional control is of central importance during haploid differentiation due to the transcriptional silencing that results from the tight packing of chromatin with protamines [2], [4]. Since the most striking defects in the differentiation of Dicer-null male germ cells coincide chronologically with chromatin condensation and transcriptional silencing, it can be envisaged that Dicer is involved in this post-transcriptional control of haploid mRNAs. High throughput transcriptome analysis as well as detailed quantitative proteomics will uncover the genes that are under the regulation of Dicer-generated small RNAs during male germ cell differentiation.

piRNAs have a well-characterized role in transposon silencing in male germ cells [40], and several studies have also linked Dicer-dependent pathways with the regulation of transposon expression [14], [15], [18], [34]. The most extensive transposon derepression in male germ cells takes place during epigenetic reprogramming in PGCs and in very early postnatal cells (from embryonic day 15 to 3 dpp), which enables the establishment of novel sex-specific epigenetic marks in the genome [40]. The level of transposable element transcripts seemed unchanged in our *Dicer1* knockout model (Fig. 7). In contrast, transposon expression was increased in the spermatocytes of *Dcr(fx/fx); Ddx4Cre* mice, which shows efficient *Dicer1* recombination already at birth (Romero et al., co-submitted manuscript). This difference may be explained by the later Cre expression in *Dcr(fx/fx); Ngn3Cre* testes only after major epigenetic reprogramming events have been largely completed. Interestingly, we observed an increase in centromeric major repeat transcript expression in *Dicer1* knockout testes (Fig. 7). Similar defects in centromeric silencing have been reported in mouse embryonic stem cells lacking functional Dicer [35], [36]. Most of the known Dicer-dependent functions in mammals involve post-transcriptional regulation, but evidence of a small RNA–mediated transcriptional gene silencing and regulation of heterochromatin formation and maintenance is emerging, even though the mechanistic aspects still remain unclear [41]. Dicer has been localized in the nucleus and specifically on certain chromosomal domains suggesting that Dicer has a role in transcriptional regulation at the chromatin level [42], [43]. However, further studies will be required to reveal the possible mechanistic connection of Dicer and Dicer-dependent small RNAs with heterochromatin formation, regulation of repeat-derived transcripts and control of chromatin organization in differentiating male germ cells.

## Materials and Methods

### Ethics statement

Animal husbandry and all animal experimentation were carried out in compliance with Finnish laws. All efforts were made to minimize the suffering of animals. Protocols for the use of animals were approved by the Committee on the Ethics of Animal Experimentation at the University of Turku in accordance with the Guide for Care and Use of Laboratory Animals (National Academy of Science) (license number: 2009-1206-Kotaja).

### Animals

Mice were housed at the Animal Facility of the University of Turku, Finland, under controlled environmental conditions. The genetic background of all the mice used in this study was mixed C57Bl/6J and SV129. Floxed *Dicer1* mice were kindly provided by B. Harfe and were genotyped as described [28]. Genotyping primers for *Ngn3Cre* mice [27] were: F(Ngn3Cre) 5′-CCTGTTTTGCACGTTCACCG-3′, R(Ngn3Cre) 5′-ATGCTTCTGTCCGTTTGCCG-3′, F(pTimer) 5′-ACGGCTGCTTCATCTACAAGG-3′, R(pTimer) 5′-TTGGTGTCCACGTAGTAGTAG-3′. To achieve selective inactivation of *Dicer1* in germ cells, transgenic *Ngn3Cre* female mice were mated with male mice carrying two floxed *Dicer1 (Dcr)* alleles in order to generate *Dcr(fx/wt); Ngn3Cre* and *Dcr(fx/wt)* mice. These animals were then intercrossed to produce *Dcr(fx/fx); Ngn3Cre* as well *Dcr(fx/fx)* and *Dcr(fx/wt); Ngn3Cre* control littermates. Recombination was confirmed with deletion-specific genomic PCR using primers described earlier [28]. The *ROSA26-YFP* reporter construct consist of a stop cassette upstream of the YFP coding sequence that is inserted in the *ROSA26* gene locus; upon Cre expression the stop cassette is removed and expression of the reporter is induced [30].

### Primary antibodies

The following rabbit polyclonal antibodies were used: anti-GFP (A11122, Invitrogen), anti-DDX4/MVH (1∶1000, from T. Noce), anti-GATA4 (1∶50, sc-9053, Santa Cruz), anti-CENP-A (1∶1000, sc-22814, Santa Cruz), anti-phosphorylated histone H3 Ser10 (1∶100, Millipore), anti-SCP3 (1∶100, sc-33195, Santa Cruz) and anti-PRM1 (1∶100, sc-30174, Santa Cruz). The following mouse monoclonal antibodies were used: anti-phosphorylated histone H2AX (1∶100, Millipore), anti-αTubulin (1∶1000, Thermo Scientific), anti-dimethylated histone H3 Lys9 (1∶500, Millipore), anti-Cre (1∶100, Covance), anti-CBX1/HP1β (1∶100, Abcam), anti-acetylated histone H3 Lys9 (1∶500, Millipore), anti-AKAP4 (1∶200, BD Biosciences), and anti-H1T2 (1∶500, from I. Davidson).

### RNA extraction and qRT-PCR

Total testis RNAs were extracted using TRI Reagent solution (Molecular Research Center, Inc.) and treated with TURBO DNase (Ambion). cDNA synthesis and qPCR quantification was performed by using DyNAmo™ cDNA synthesis kit (Finnzymes) and DyNAmo™ Flash SYBR® Green qPCR Kit (Finnzymes). Primers for *Dicer1* were: F(Dicer) 5′-CTTGACTGACTTGCGCTCTG-3′ and R(Dicer) 5′-AATGGCACCAGCAAGAGACT-3′. Primers for SINE B1 and LINE L1 RNA are described in [44] and IAP primers are described in [45]. Primers for centromeric major and minor repeat transcripts are described in [46]. Normalization was performed with L19 mRNA levels for repetitive elements and mRNA transcripts. Primers for L19 were: F(L19) 5′-GGACAGAGTCTTGATGATCTC-3′ and R(L19) 5′-CTGAAGGTCAAAGGGAATG-TG-3′. Each assay was performed in three independent technical and biological replicates.

### Histology

For histological analyses, tissues were collected and directly fixed in 4% paraformaldehyde (PFA) or in Bouin's fixative (4–20 hours at room temperature). Tissues were then dehydrated in a series of ethanol washes and embedded in paraffin. Paraffin-embedded tissues were cut and stained with hematoxylin and eosin (HE) or periodic acid-Schiff (PAS) according to standard protocols.

### Flow cytometric analysis

Cell suspensions prepared from *Dcr(fx/fx)*, *Dcr(fx/wt); Ngn3Cre* and *Dcr(fx/fx); Ngn3Cre* testes of 1-year old mice were separated on a FACScalibur machine according to their DNA content, that is haploid (1C), diploid (2C) or tetraploid (4C). FACS analysis was performed as described earlier [47].

### Squash preparations and drying down preparations

Testes were dissected and decapsulated, and the specific stages of the seminiferous epithelial cycle of corresponding tubule segments were identified on the basis of their transillumination patterns. Squash preparations were prepared according to the protocol in [31], and live cell microscopy was performed by using phase contrast optics (Leica DMRBE microscope). For drying down preparations, stage-specific segments of seminiferous tubules were isolated, and cells were released and fixed on slides as previously described [31]. Preparations were visualized by phase contrast microscopy or used for immunofluorescence.

### Immunohistochemistry and immunofluorescence

Paraffin-embedded testis sections were rehydrated, antigens were retrieved by pressure cooking in 10 mM Sodium Citrate (pH 6.5) for 2 hours, and non-specific binding sites were blocked in 3% BSA, 10% normal goat serum. Endogenous peroxidase activity was blocked in 3% H_2_O_2_. Antibody incubations were done in blocking solution for 1 hour at 37°C, or O/N at 4°C. Rabbit and mouse IgG were used as negative controls (Vector Laboratories). For histochemical detection, tissue sections were incubated with a biotinylated secondary antibody (1∶750, Vector Laboratories) diluted in blocking solution for 1 hour at 37°C. Antibody localization was detected with the regular VECTASTAIN ABC (peroxidase) system (Vector Laboratories) and 3,3′-diaminobenzidine (liquid DAB+, DAKO), stained with hematoxylin, dehydrated and mounted in PERTEX medium. Alternatively, AlexaFuor488 and AlexaFluor594 conjugated secondary antibodies (1∶500, Invitrogen) diluted in blocking solution were used for immunofluorescence detection. Nuclei were stained with 4′,6-diamidino-2-phenylindole (DAPI) (1∶10 000, Sigma) for 5 minutes and sections were mounted in Mowiol 4–88 medium (Polysciences, Inc.). Drying down and squash preparations were post-fixed in 4% paraformaldehyde, permeabilized in 0.2% Triton-X 100 for 15 minutes at room temperature, and immunofluorescence was performed as described above. For the detection of mitochondria, slides were incubated with 200 nM Mitotracker (Invitrogen) in PBS for 15 minutes. Zeiss AxioImager M1 microscope was used for the normal immunofluoresence analyses. Zeiss LSM510 META microscope was used for the confocal images.

### TUNEL assay

TdT-mediated dUTP nick end labelling (TUNEL) was used on Bouin's fixed, paraffin embedded testis sections to detect DNA fragmentation in apoptotic cells. Paraffin was removed and sections were rehydrated and permeabilized in 10 mM Sodium Citrate (pH 6.5) for 20 minutes in a microwave. Endogenous peroxidase activity was blocked in 3% H_2_O_2_ (Sigma) at room temperature for 15 minutes. Sections were incubated in 1× TdT buffer, Terminal transferase (0.6 U, Roche), biotin-16-dUTP (10 µM, Roche) and 10 µM CoCl_2_ for an hour at 37°C. A negative control was incubated without an enzyme. Non-specific sites were blocked with 3% BSA and 10% Normal Goat Serum for 15 minutes at room temperatures and subsequently incubated with ExtrAvidin®–Peroxidase (1∶50, Sigma) for 30 minutes at 37°C. Apoptotic cells were detected with 3,3′-diaminobenzidine (liquid DAB+, DAKO), and cells were stained with hematoxylin, dehydrated and mounted with PERTEX medium.

### Sperm counts and sperm slides

Epididymal sperm count was performed with sperm extracted from the cauda epididymis and ductus deferens of adult (60 dpp or 120 dpp) male mice and analyzed for its concentration as previously described [48]. Epididymal sperm released in PBS from cauda epididymides was spread on glass slides, air dried and either stained with hematoxylin for morphological analysis or used for immunofluorescence.

### Electron microscopy

Testis and epididymal sperm samples were fixed in 5% glutaraldehyde and treated with a potassium ferrocyanide-osmium fixative. The samples were embedded in epoxy resin (Glycidether 100, Merck), sectioned, stained with 5% uranyl acetate and 5% lead citrate, and visualized on a JEOL 1200 EX transmission electron microscope.

## Supporting Information

Figure S1 **HE staining of testis sections.** Bouin's-fixed and paraffin-embedded *Dcr(fx/fx)* and *Dcr(fx/fx);Ngn3Cre* testes section at 21 and 35 dpp were stained by hematoxylin and eosin. Staging of the seminiferous epithelial cycle was done on the basis of the presence and organization of different types of spermatogonia, and on the basis of the presence, organization and size of spermatocytes. Stages of the seminiferous epithelial cycle are indicated in the lumen of each tubule cross-section. Scale bar: 25 µm.
(TIF)

Figure S2 **PAS staining of adult testis sections.** Bouin's-fixed and paraffin embedded *Dcr(fx/fx)* and *Dcr(fx/fx);Ngn3Cre* testes were sectioned and stained with Periodic-Acid-Schiff. Staging of the seminiferous epithelial cycle of the tubule cross sections was done on the basis of the presence and organization of different types of spermatids. Stages of the seminiferous epithelial cycle are indicated in the upper left corner of each image. Scale bar: 25 µm.
(TIF)

Figure S3 **Tubular organization in knockout testes is normal.** Anti-DDX4/MVH (A) and anti-GATA4 (B) immunofluorescence on *Dcr(fx/fx)*, *Dcr(fx/wt);Ngn3Cre* and *Dcr(fx/fx);Ngn3Cre* testis sections (60 dpp and 120 dpp) revealed the normal localization of germ (DDX4/MVH) and Sertoli (GATA4) cells within the heterozygous and knockout seminiferous tubules. Scale bar: 50 µm.
(TIF)

Figure S4 **Electron microscopic analysis.** A) Synaptonemal complexes (arrows) were detected in knockout pachytene spermatocytes. B) Fragmented acrosomes and abnormal acrosomal structures were frequently observed (asterisk), and Golgi complexes appeared unusually prominent (arrows) in knockout round spermatids. C) The chromatoid body (arrows) of *Dicer1* knockout round spermatids did not have any gross abnormalities. D) Step 9 elongating spermatids appeared affected and the polarization of the nucleus in the apical side of the cell was often lost. Scale bar: 2 µm. White small round wholes throughout the preparations are artefacts from sample processing.
(TIF)

Figure S5 **Heterochromatin patterns in** ***Dicer1*** **knockout male germ cells.** Immunofluorescence staining of control and knockout testis sections with antibodies against dimethylated H3 Lysine 9 (A) and CENP-A (B) demonstrated no gross abnormalities in the heterochromatin patterns of knockout testes. Nuclei are stained with DAPI (blue). Stages of the seminiferous epithelial cycle are indicated for each tubule. Scale bar: 50 µm.
(TIF)

## References

[1] Kimmins S, Kotaja N, Davidson I, Sassone-Corsi P (2004). Testis-specific transcription mechanisms promoting male germ-cell differentiation.. Reproduction.

[2] Kimmins S, Sassone-Corsi P (2005). Chromatin remodelling and epigenetic features of germ cells.. Nature.

[3] Hess RA, Renato de Franca L (2008). Spermatogenesis and cycle of the seminiferous epithelium.. Adv Exp Med Biol.

[4] Gaucher J, Reynoird N, Montellier E, Boussouar F, Rousseaux S (2010). From meiosis to postmeiotic events: the secrets of histone disappearance.. FEBS J.

[5] Ghildiyal M, Zamore PD (2009). Small silencing RNAs: an expanding universe.. Nat Rev Genet.

[6] Lau NC (2010). Small RNAs in the animal gonad: guarding genomes and guiding development.. Int J Biochem Cell Biol.

[7] Ro S, Park C, Sanders KM, McCarrey JR, Yan W (2007). Cloning and expression profiling of testis-expressed microRNAs.. Dev Biol.

[8] Chiang HR, Schoenfeld LW, Ruby JG, Auyeung VC, Spies N (2010). Mammalian microRNAs: experimental evaluation of novel and previously annotated genes.. Genes Dev.

[9] Guo H, Ingolia NT, Weissman JS, Bartel DP (2010). Mammalian microRNAs predominantly act to decrease target mRNA levels.. Nature.

[10] Friedman RC, Farh KK, Burge CB, Bartel DP (2009). Most mammalian mRNAs are conserved targets of microRNAs.. Genome Res.

[11] Siomi MC, Sato K, Pezic D, Aravin AA (2011). PIWI-interacting small RNAs: the vanguard of genome defence.. Nat Rev Mol Cell Biol.

[12] Aravin AA, Hannon GJ, Brennecke J (2007). The Piwi-piRNA pathway provides an adaptive defense in the transposon arms race.. Science.

[13] Krol J, Loedige I, Filipowicz W (2010). The widespread regulation of microRNA biogenesis, function and decay.. Nat Rev Genet.

[14] Tam OH, Aravin AA, Stein P, Girard A, Murchison EP (2008). Pseudogene-derived small interfering RNAs regulate gene expression in mouse oocytes.. Nature.

[15] Watanabe T, Totoki Y, Toyoda A, Kaneda M, Kuramochi-Miyagawa S (2008). Endogenous siRNAs from naturally formed dsRNAs regulate transcripts in mouse oocytes.. Nature.

[16] Song R, Hennig GW, Wu Q, Jose C, Zheng H (2011). Male germ cells express abundant endogenous siRNAs.. Proc Natl Acad Sci U S A.

[17] Bernstein E, Kim SY, Carmell MA, Murchison EP, Alcorn H (2003). Dicer is essential for mouse development.. Nat Genet.

[18] Murchison EP, Stein P, Xuan Z, Pan H, Zhang MQ (2007). Critical roles for Dicer in the female germline.. Genes Dev.

[19] Tang F, Kaneda M, O'Carroll D, Hajkova P, Barton SC (2007). Maternal microRNAs are essential for mouse zygotic development.. Genes Dev.

[20] Hayashi K, Chuva de Sousa Lopes SM, Kaneda M, Tang F, Hajkova P (2008). MicroRNA biogenesis is required for mouse primordial germ cell development and spermatogenesis.. PLoS One.

[21] Maatouk DM, Loveland KL, McManus MT, Moore K, Harfe BD (2008). Dicer1 is required for differentiation of the mouse male germline.. Biol Reprod.

[22] Papaioannou MD, Pitetti JL, Ro S, Park C, Aubry F (2009). Sertoli cell Dicer is essential for spermatogenesis in mice.. Dev Biol.

[23] Papaioannou MD, Lagarrigue M, Vejnar CE, Rolland AD, Kuhne F (2011). Loss of Dicer in Sertoli cells has a major impact on the testicular proteome of mice.. Mol Cell Proteomics.

[24] Lomeli H, Ramos-Mejia V, Gertsenstein M, Lobe CG, Nagy A (2000). Targeted insertion of Cre recombinase into the TNAP gene: excision in primordial germ cells.. Genesis.

[25] Kotaja N, Bhattacharyya SN, Jaskiewicz L, Kimmins S, Parvinen M (2006). The chromatoid body of male germ cells: similarity with processing bodies and presence of Dicer and microRNA pathway components.. Proc Natl Acad Sci U S A.

[26] Gonzalez-Gonzalez E, Lopez-Casas PP, del Mazo J (2008). The expression patterns of genes involved in the RNAi pathways are tissue-dependent and differ in the germ and somatic cells of mouse testis.. Biochim Biophys Acta.

[27] Desgraz R, Herrera PL (2009). Pancreatic neurogenin 3-expressing cells are unipotent islet precursors.. Development.

[28] Harfe BD, McManus MT, Mansfield JH, Hornstein E, Tabin CJ (2005). The RNaseIII enzyme Dicer is required for morphogenesis but not patterning of the vertebrate limb.. Proc Natl Acad Sci U S A.

[29] Yoshida S, Takakura A, Ohbo K, Abe K, Wakabayashi J (2004). Neurogenin3 delineates the earliest stages of spermatogenesis in the mouse testis.. Dev Biol.

[30] Srinivas S, Watanabe T, Lin CS, William CM, Tanabe Y (2001). Cre reporter strains produced by targeted insertion of EYFP and ECFP into the ROSA26 locus.. BMC Dev Biol.

[31] Kotaja N, Kimmins S, Brancorsini S, Hentsch D, Vonesch JL (2004). Preparation, isolation and characterization of stage-specific spermatogenic cells for cellular and molecular analysis.. Nat Methods.

[32] Hazzouri M, Pivot-Pajot C, Faure AK, Usson Y, Pelletier R (2000). Regulated hyperacetylation of core histones during mouse spermatogenesis: involvement of histone deacetylases.. Eur J Cell Biol.

[33] Martianov I, Brancorsini S, Catena R, Gansmuller A, Kotaja N (2005). Polar nuclear localization of H1T2, a histone H1 variant, required for spermatid elongation and DNA condensation during spermiogenesis.. Proc Natl Acad Sci U S A.

[34] Kaneko H, Dridi S, Tarallo V, Gelfand BD, Fowler BJ (2011). DICER1 deficit induces Alu RNA toxicity in age-related macular degeneration.. Nature.

[35] Kanellopoulou C, Muljo SA, Kung AL, Ganesan S, Drapkin R (2005). Dicer-deficient mouse embryonic stem cells are defective in differentiation and centromeric silencing.. Genes Dev.

[36] Murchison EP, Partridge JF, Tam OH, Cheloufi S, Hannon GJ (2005). Characterization of Dicer-deficient murine embryonic stem cells.. Proc Natl Acad Sci U S A.

[37] Song J, Xu Y, Hu X, Choi B, Tong Q (2010). Brain expression of Cre recombinase driven by pancreas-specific promoters.. Genesis.

[38] Catena R, Ronfani L, Sassone-Corsi P, Davidson I (2006). Changes in intranuclear chromatin architecture induce bipolar nuclear localization of histone variant H1T2 in male haploid spermatids.. Dev Biol.

[39] Martianov I, Brancorsini S, Gansmuller A, Parvinen M, Davidson I (2002). Distinct functions of TBP and TLF/TRF2 during spermatogenesis: requirement of TLF for heterochromatic chromocenter formation in haploid round spermatids.. Development.

[40] Castaneda J, Genzor P, Bortvin A (2011). piRNAs, transposon silencing, and germline genome integrity..

[41] Moazed D (2009). Small RNAs in transcriptional gene silencing and genome defence.. Nature.

[42] Khalil AM, Driscoll DJ (2010). Epigenetic regulation of pericentromeric heterochromatin during mammalian meiosis.. Cytogenet Genome Res.

[43] Sinkkonen L, Hugenschmidt T, Filipowicz W, Svoboda P (2010). Dicer is associated with ribosomal DNA chromatin in mammalian cells.. PLoS One.

[44] Martens JH, O'Sullivan RJ, Braunschweig U, Opravil S, Radolf M (2005). The profile of repeat-associated histone lysine methylation states in the mouse epigenome.. EMBO J.

[45] Rowe HM, Jakobsson J, Mesnard D, Rougemont J, Reynard S (2010). KAP1 controls endogenous retroviruses in embryonic stem cells.. Nature.

[46] Lehnertz B, Ueda Y, Derijck AA, Braunschweig U, Perez-Burgos L (2003). Suv39h-mediated histone H3 lysine 9 methylation directs DNA methylation to major satellite repeats at pericentric heterochromatin.. Curr Biol.

[47] Malkov M, Fisher Y, Don J (1998). Developmental schedule of the postnatal rat testis determined by flow cytometry.. Biol Reprod.

[48] Guerif F, Cadoret V, Plat M, Magistrini M, Lansac J (2002). Characterization of the fertility of Kit haplodeficient male mice.. Int J Androl.

