# Supplementary figures and images for: Dicer Is Required for Haploid Male Germ Cell Differentiation in Mice
Source: PLoS One. 2011 Sep 16;6(9):e24821. doi: 10.1371/journal.pone.0024821 (PMC3174967; doi:10.1371/journal.pone.0024821)

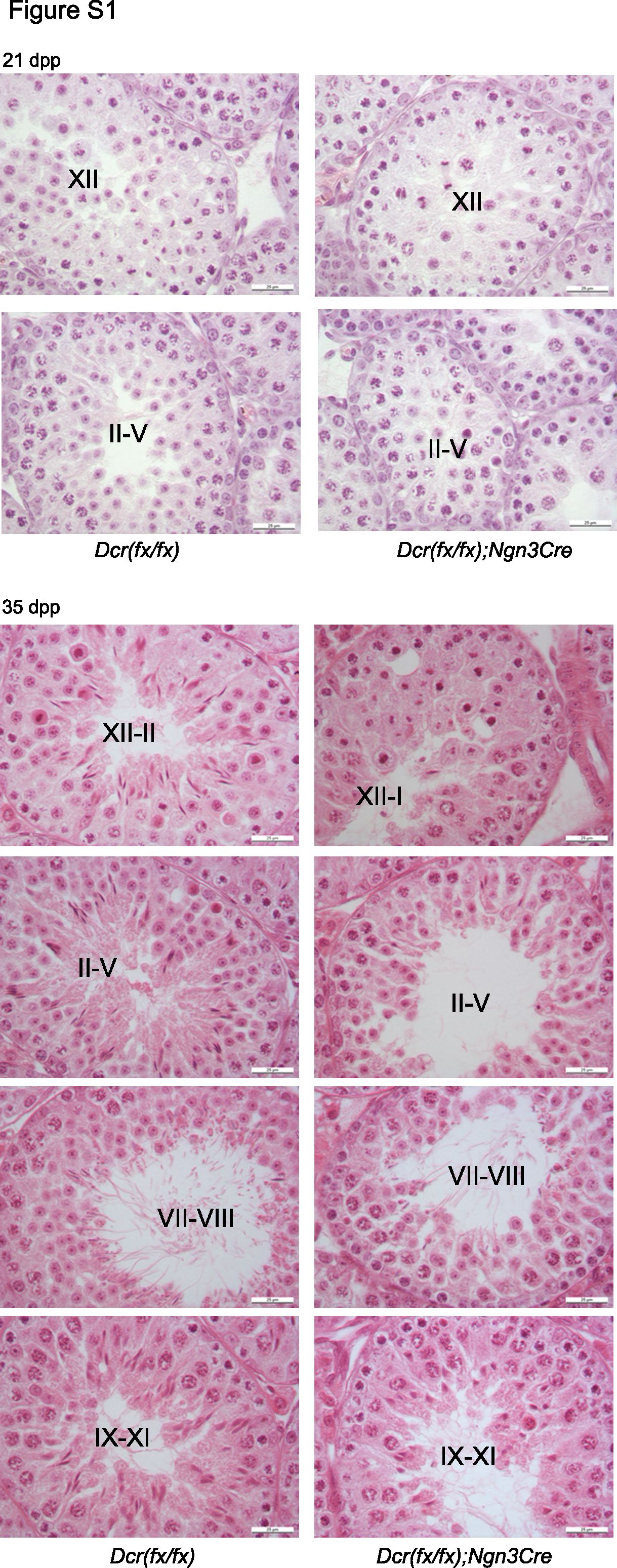

Supplement: Figure S1 — HE staining of testis sections. Bouin's-fixed and paraffin-embedded Dcr(fx/fx) and Dcr(fx/fx);Ngn3Cre testes section at 21 and 35 dpp were stained by hematoxylin and eosin. Staging of the seminiferous epithelial cycle was done on the basis of the presence and organization of different types of spermatogonia, and on the basis of the presence, organization and size of spermatocytes. Stages of the seminiferous epithelial cycle are indicated in the lumen of each tubule cross-section. Scale bar: 25 µm. (TIF) [file pone.0024821.s001.tif]

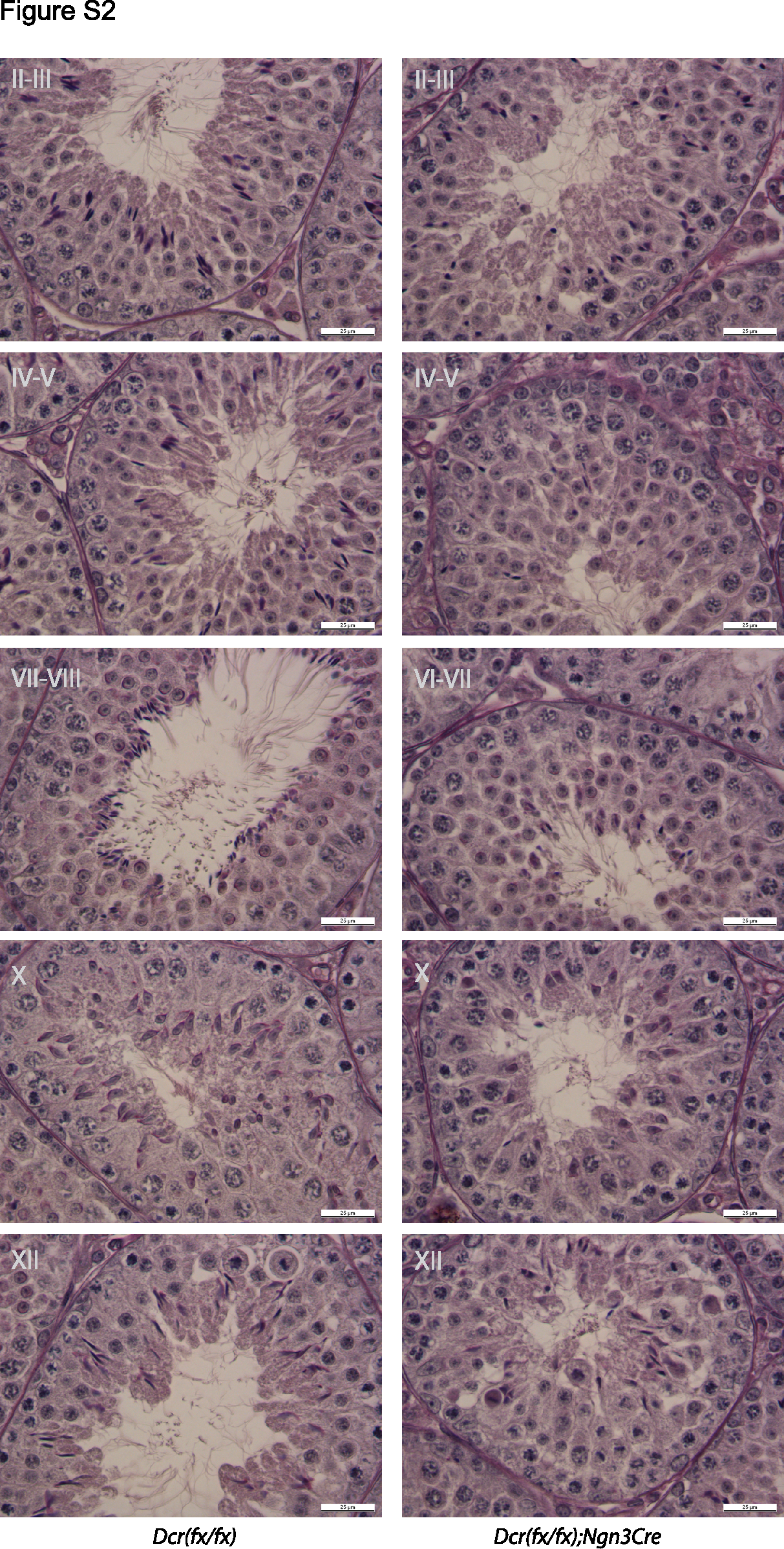

Supplement: Figure S2 — PAS staining of adult testis sections. Bouin's-fixed and paraffin embedded Dcr(fx/fx) and Dcr(fx/fx);Ngn3Cre testes were sectioned and stained with Periodic-Acid-Schiff. Staging of the seminiferous epithelial cycle of the tubule cross sections was done on the basis of the presence and organization of different types of spermatids. Stages of the seminiferous epithelial cycle are indicated in the upper left corner of each image. Scale bar: 25 µm. (TIF) [file pone.0024821.s002.tif]

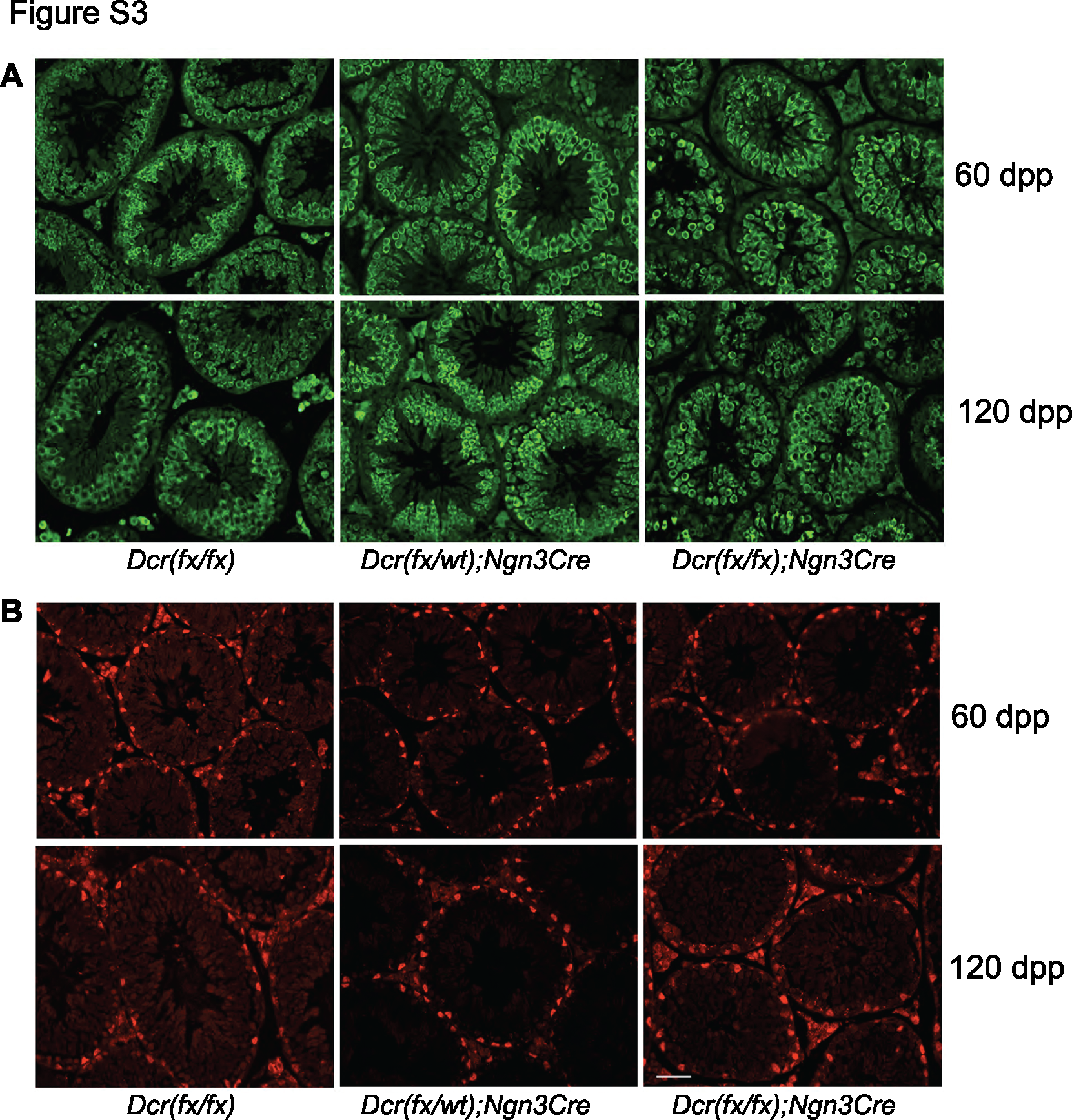

Supplement: Figure S3 — Tubular organization in knockout testes is normal. Anti-DDX4/MVH (A) and anti-GATA4 (B) immunofluorescence on Dcr(fx/fx), Dcr(fx/wt);Ngn3Cre and Dcr(fx/fx);Ngn3Cre testis sections (60 dpp and 120 dpp) revealed the normal localization of germ (DDX4/MVH) and Sertoli (GATA4) cells within the heterozygous and knockout seminiferous tubules. Scale bar: 50 µm. (TIF) [file pone.0024821.s003.tif]

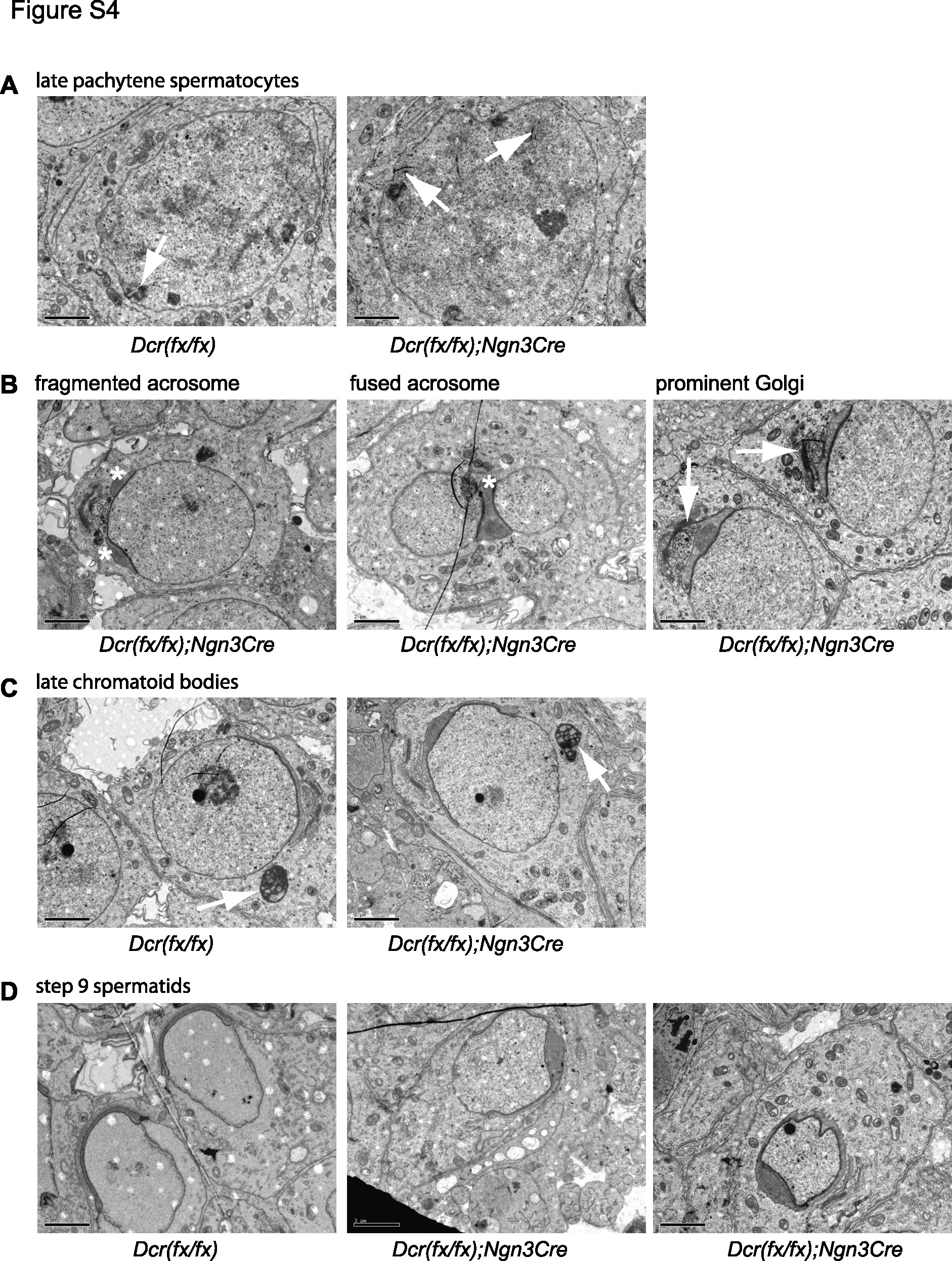

Supplement: Figure S4 — Electron microscopic analysis. A) Synaptonemal complexes (arrows) were detected in knockout pachytene spermatocytes. B) Fragmented acrosomes and abnormal acrosomal structures were frequently observed (asterisk), and Golgi complexes appeared unusually prominent (arrows) in knockout round spermatids. C) The chromatoid body (arrows) of Dicer1 knockout round spermatids did not have any gross abnormalities. D) Step 9 elongating spermatids appeared affected and the polarization of the nucleus in the apical side of the cell was often lost. Scale bar: 2 µm. White small round wholes throughout the preparations are artefacts from sample processing. (TIF) [file pone.0024821.s004.tif]

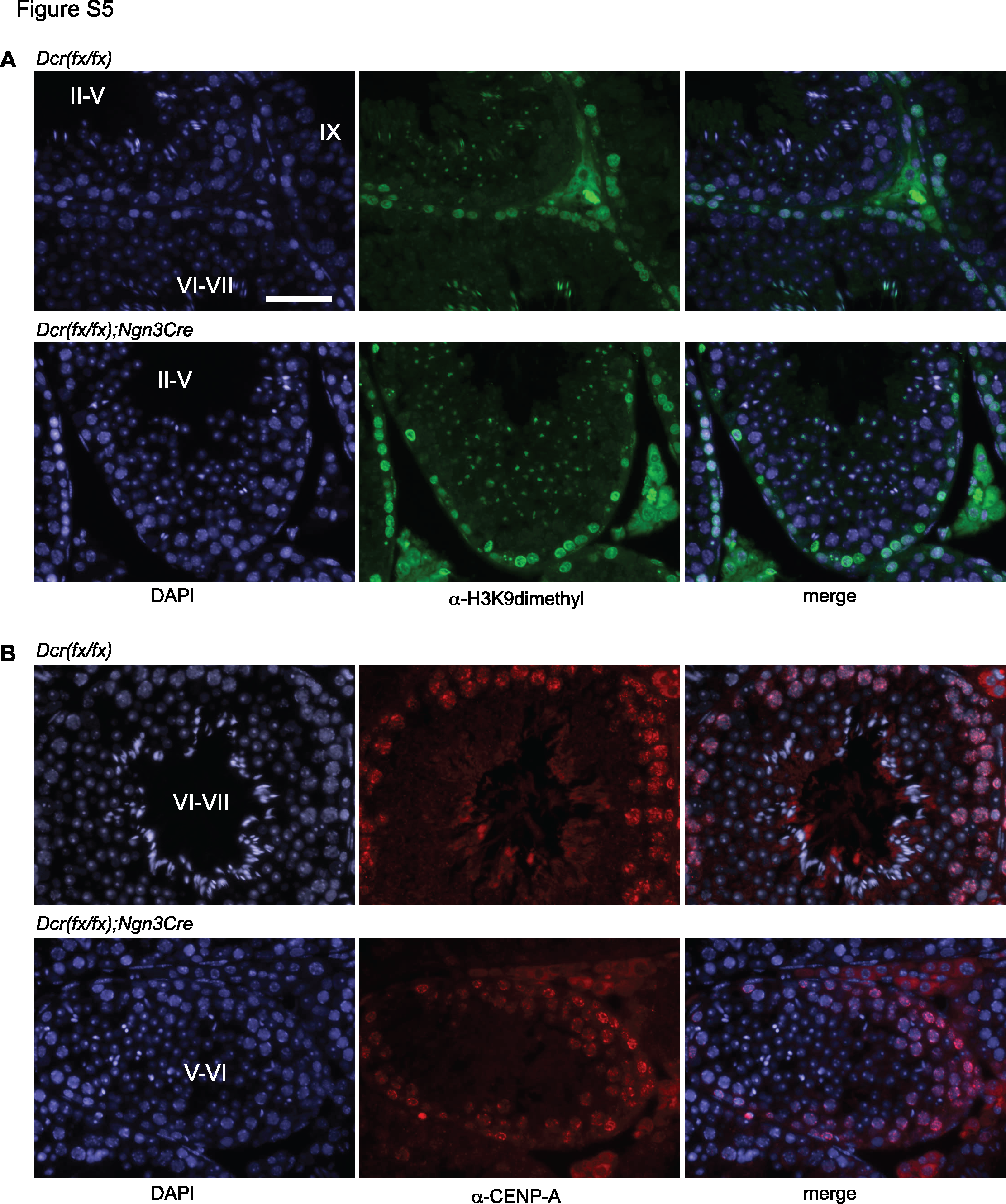

Supplement: Figure S5 — Heterochromatin patterns in Dicer1 knockout male germ cells. Immunofluorescence staining of control and knockout testis sections with antibodies against dimethylated H3 Lysine 9 (A) and CENP-A (B) demonstrated no gross abnormalities in the heterochromatin patterns of knockout testes. Nuclei are stained with DAPI (blue). Stages of the seminiferous epithelial cycle are indicated for each tubule. Scale bar: 50 µm. (TIF) [file pone.0024821.s005.tif]
